# Executive Dysfunction in Patients With Alcohol Use Disorder: A Systematic Review

**DOI:** 10.7759/cureus.29207

**Published:** 2022-09-15

**Authors:** Shrinkhala Maharjan, Zainab Amjad, Abdelrahman Abaza, Advait M Vasavada, Akhil Sadhu, Carla Valencia, Hameeda Fatima, Ijeoma Nwankwo, Mahvish Anam, Lubna Mohammed

**Affiliations:** 1 Internal Medicine, California Institute of Behavioral Neurosciences & Psychology, Fairfield, USA; 2 Internal Medicine, College of Medicine, King Faisal University, Al Ahsa, SAU; 3 Pathology, California Institute of Behavioral Neurosciences & Psychology, Fairfield, USA; 4 Internal Medicine, M.P. Shah Medical College, Jamnagar, IND; 5 Emergency Medicine and Plastic Surgery, All India Institute of Medical Sciences, New Delhi, IND; 6 Family Medicine, California Institute of Behavioral Neurosciences & Psychology, Fairfield, USA; 7 Research, California Institute of Behavioral Neurosciences & Psychology, Fairfield, USA; 8 Internal Medicine, Dow Medical College, Dow University of Health Sciences, Dr. Ruth K. M. Pfau Civil Hospital Karachi, Karachi, PAK

**Keywords:** cognitive impairment, executive dysfunction, neurophysiology, executive function, alcohol use disorder

## Abstract

A medical condition known as alcohol use disorder (AUD) is defined as an impaired capacity to reduce or regulate alcohol consumption despite negative social, occupational, or health effects. According to studies, habitual drinkers experience a reduction in their capacity to process new information, gain new skills, and formulate plans. Studies indexed in PubMed, PubMed Central, Google Scholar, ResearchGate, and ScienceDirect, published from 2012 to 2022, were identified through the search terms “alcohol use disorder” and “executive function.” A total of 2242 abstracts were identified through the initial search terms. Full texts were reviewed for 61 articles, out of which nine articles met the criteria for inclusion. This systematic review was based on the Preferred Reporting Items for Systematic Reviews and Meta-Analyses (PRISMA) guidelines. The current systematic review primarily focuses on the following issues: clinical neuropsychological tests of executive dysfunction, specific brain regions most affected by alcohol neurotoxic effects, and alcohol-related dementia. This review concluded that chronic alcohol dependence syndrome causes impairments in several cognitive function domains. Study shows frontal lobe damage is caused by chronic alcohol consumption. A faulty interaction among large-scale networks underlies patients' executive dysfunction in AUD, which is suggested by changes in prefrontal white-matter pathways. The goal of this systematic review is to improve the ability to recognize alcoholics who are particularly at risk of functional impairments to tailor therapeutic therapy to maximize the chance of maintaining abstinence and neuropsychology concerning this complex disease.

## Introduction and background

Alcohol use disorder (AUD) is a relapsing chronic brain disease marked by excessive alcohol consumption despite negative consequences [1]. With almost 3.3 million fatalities each year (5.9% of all deaths), it continues to be a severe burden in the majority of the world's nations [1]. Studies reveal that chronic alcoholics have impairments, such as a decline in their capacity to process new information, pick up new skills, and formulate strategies [2,3]. Several studies have measured the decrease in neurocognitive function in chronic alcoholics [2].

A higher-order cognitive concept known as executive functioning (EF) is engaged in the self-regulation of goal-directed behavior [4]. It describes a broad range of cognitive skills, such as sustained and selective attention, mental flexibility, response inhibition, supervisory control of action, and interference resistance [4]. According to one definition, executive functions consist of four parts: the capacity to create goals, plan how to reach those goals, follow the plans, and execute well. Executive dysfunction is also linked to episodic memory deficits brought on by chronic drinking [2].

Long-term studies have shown that cognitive deficits in several areas, such as visuospatial processing, memory, and EF, are related to AUD. About half of AUD patients have cognitive deficiencies, which can have a substantial impact on their treatment compliance and day-to-day functioning, with EF playing a critical part in this process [3]. Cognitive impairments are present in between 30% and 80% of those seeking treatment for AUD [5]. It is significant in people with Korsakoff's syndrome (KS) and is a defining feature of the condition. But most individuals with alcohol-related cognitive impairments (ARCI) do not meet the requirements for KS because they have less severe cognitive abnormalities that are frequently ignored and under-diagnosed by physicians [5]. Alcohol addiction causes poor medical, familial, and social outcomes and affects the overall quality of life of the patient, leading to homelessness, unemployment, and, particularly, mental health disorders, which are essential topics for health professionals [6].

Alcohol use disorder and its causes

According to Koob and colleagues, the addiction cycle has three stages: the binge and intoxication stage, the withdrawal stage, and the fixation and anticipation stage. The reward system is initially hyperactive during the binge and intoxication stages, particularly the ventral tegmentum and the nucleus accumbens, and after prolonged alcohol consumption, a homeostatic change to hypoactivation takes place. Focus is placed on decreased feelings of reward for traditional stimuli and increases in negative affect during withdrawal and negative affect. Changes in the functioning of the striatum, extended amygdala, and insula accompany these psychological events. Finally, the third stage is impacted by stress, results in greater disinhibition, obsession with reward anticipation, including desire, and raises the chance of relapse [6].

In each stage of the addiction process, including drug acquisition, escalation/dysregulation, abstinence, and relapse, the decisional and cognitive components of impulsivity are crucial [3]. Its use is likely to start as a result of social, religious, and psychological influences. Genetic studies in the recent past have given credence to the biological aspect of addiction [6]. Low conscientiousness, low agreeableness, and high neuroticism are three of the five personality factors that have significant cross-sectional and longitudinal associations with various alcohol outcomes, including consumption and alcohol-related problems and disorders [7].

The advancement of neuroscience has made it possible to conceptualize addiction as a chronic brain illness that includes a variety of elements, including sociocultural, genetic, and even neurodevelopmental characteristics [8]. Awareness of the causes, impact, and treatment of AUDs requires understanding individual variability in EF [9]. Patients abusing alcohol are more prone to developing AUD and exhibit specific psychological traits, such as high behavioral activation, low self-esteem, low family function, and low life satisfaction [6].

The National Institute on Alcohol Abuse and Alcoholism (NIAAA) and the World Health Organization (WHO) have separated alcohol usage into five categories. These categories include (1) long-term abstinence from alcohol (LA); (2) binge drinking (BD), which is defined as consuming a large amount of alcohol in a short period (within two hours on one occasion) without any intention of stopping (two to three times per month, of more than equals to four drinks for females and five drinks for men); (3) long-term damaging alcohol consumption (LD), which is defined as exceeding the safe drinking dosage advised by the WHO, with alcohol intake of three to four drinks for females and four to five drinks for males; (4) long-term low dose drinking (LDD), which involves consuming no more than two drinks of alcohol per day for both sexes; (5) long-term heavy drinking (HD), which involves consuming more than five drinks per day of alcohol, regardless of sex [10].

Changes in neuroanatomy

Despite widespread agreement on the effects of alcohol on cognitive function, various hypotheses about the underlying neurocognitive mechanisms have been advanced. Chronic alcohol consumption affects the connections as well as the morphology of the brain. The diencephalic, hippocampus, and frontal regions are most affected by widespread patterns of macro and micro-structural alterations [11]. The prominent executive impairment seen in AUDs may reflect the frontal cortex's particular susceptibility. However, neuroimaging morphometric studies have shown more extensive damage in frontal and cerebellar regions, limbic structures, and the basal ganglia, supporting the diffuse brain hypothesis [1,12,13].

Analysis of resting-state brain activity, or the intrinsic level of neural function involving spontaneous slow fluctuations of the blood-oxygenation-level-dependent (BOLD) signal, may provide pertinent information. This method reveals functionally connected and temporally coherent networks that overlap with those linked to sensory, cognitive, or motor performance, even without external stimulation. This equivalence suggests that the neurophysiological processes underlying individual differences in cognitive functioning have a baseline activity in resting-state networks [1].

According to imaging studies, those who suffer from executive dysfunction and alcohol dependency have less gray matter (GM) in their prefrontal cortex [6,13]. The mesocorticolimbic pathway, i.e., subcortical (the ventral striatum, thalamus, hippocampus, and amygdala) and cortical (the ventromedial and posterior dorsomedial frontal cortex) components, showed a diffuse pattern of GM decrease in patients, according to the results of voxel-based morphometry (VBM) studies. Dysfunction of the mesocorticolimbic pathway has been proposed to underlie the emergence and maintenance of addiction through negative reinforcement, based on functional magnetic resonance imaging (fMRI) evidence for its function in adaptive behavioral learning [6,13]. In AUD patients, there is evidence of aberrant intrinsic activity, which reflects intra-network connection and coactivation levels [1].

Clinical neuropsychological tests of executive dysfunction

To find cognitive abnormalities in AUD patients, cognitive screens that assess cognitive function are performed [5]. The test-level data were divided into the following five subcategories: planning, problem-solving, reasoning and abstraction, flexibility and set-shifting, verbal fluency, and inhibition and self-control in accordance with other recent meta-analyses of EF and recognition of the interconnected yet distinct components of EF. Although they are widely employed in clinical neuropsychological evaluations to reflect the major categories under which frequently used standardized neuropsychological tests are organized, the five EF elements listed above are neither mutually exclusive nor exhaustive [3].

The Wisconsin Card Sorting Test (WCST) measures "set-shifting," or the capacity to show flexibility in the face of shifting reinforcement schedules. Utilizing environmental feedback to shift cognitive sets, strategic planning, organized searching, directing behavior toward achieving a goal, and modulating impulsive responding are just a few examples of "frontal" lobe functions that the WCST assesses. The executive function uses WCST as a gauge, due to its reported sensitivity to frontal lobe dysfunction [3,14,15].

Using single-photon emission computed tomography (SPECT), functional neuroimaging studies have found that chronic alcoholics have decreased cerebral perfusion in the frontal regions [15]. Two imaging studies provided additional proof of frontal lobe involvement in alcoholism. A positron emission tomography (PET) study found GM shrinkage in the frontostriatal, frontocerebellar, and frontolimbic circuit nodes, including the frontal and cerebellar cortices, cingulate gyrus, thalamus, and hippocampus [12].

The WHO coined the phrase "less alcohol, the better" to encourage young adults to drink less and avoid the repercussions that come with it [10]. This section covers multiple EF impairments in AUD, relationships between regional GM volumes and cognitive impairment, and lobar abnormalities in AUD. The goal of this study was to find out if people with alcohol dependence syndrome have executive function deficits.

## Review

Methods and materials

According to the Preferred Reporting Items for Systematic Reviews and Meta-Analyses (PRISMA) 2020 guidelines, this systematic review was carried out, with studies that met the review criteria being assessed and included in the study [16].

Databases and Search Strategy

A methodical search was carried out using PubMed, PubMed Central, and Google Scholar. The field search used in the process was selected based on the keywords used in the previous literature and through Medical Subject Headings (MESH), depending on the database used. Table 1 depicts databases and search strategies.

**Table 1:** Databases and search strategy

Databases	Keywords	Search strategy	Filters	Search results
PubMed	Alcoholic, alcohol use disorder, executive function, neurophysiology, executive control network, cognitive impairment	Executive function or ("Executive Function/classification"[Mesh]) AND neurophysiology or ("Neurophysiology/classification"[Mesh]) AND alcohol use disorder or ("Alcohol-Induced Disorders/classification"[Mesh]) OR "Alcohol-Induced Disorders/complications"[Mesh] OR "Alcohol-Induced Disorders/diagnosis"[Mesh] OR "Alcohol-Induced Disorders/pathology"[Mesh] OR "Alcohol-Induced Disorders/physiopathology"[Mesh] OR "Alcohol-Induced Disorders/psychology"[Mesh]	Free full text, clinical trial, meta-analysis, randomized control trial, review, systematic review, in last 10 years, human studies, English only	200
PubMed Central	Alcohol use disorder, alcohol dependence, executive function, self-regulation, planning, inhibition, impulsivity, clinical neuropsychology	Executive function or ("Executive Function/classification"[Mesh]) AND neurophysiology or ("Neurophysiology/classification"[Mesh]) AND alcohol use disorder or ("Alcohol-Induced Disorders/classification"[Mesh]) OR "Alcohol-Induced Disorders/complications"[Mesh] OR "Alcohol-Induced Disorders/diagnosis"[Mesh] OR "Alcohol-Induced Disorders/pathology"[Mesh] OR "Alcohol-Induced Disorders/physiopathology"[Mesh] OR "Alcohol-Induced Disorders/psychology"[Mesh]	Open access, 10 years	45
ResearchGate	Alcohol dependence, executive dysfunction	“Alcohol dependence” AND “executive dysfunction”		9
ScienceDirect	Alcohol use disorders, resting-state fMRI, executive control network, salience network, functional connectivity	“A study of” “executive dysfunction” AND “alcohol use disorder”	2012-2022, review articles, research articles, psychiatry research, neuroscience and behavioral reviews, psychology, neuroscience	638
Google Scholar	Alcohol use disorders, executive control network, functional connectivity	“Executive dysfunction” AND “alcohol use disorder”	2012-2022, review articles	1350

Google Sheets 2022 was used to group every reference to eliminate duplication. The full-text papers were then retrieved after the records were first examined based on the titles and abstracts, and any irrelevant studies were omitted.

Inclusion and Exclusion Criteria

To determine the executive dysfunction in AUD patients who were diagnosed based on the Diagnostic and Statistical Manual of Mental Disorders, Fifth Edition (DSM-5) criteria and were at least 16 years old and under 60 years old, a systematic review was done. Electronic databases PubMed, PubMed Central, Google Scholar, ScienceDirect, and Cochrane Library were searched for English language papers published during the previous 10 years containing free full-text articles to find pertinent literature. The studies chosen were cross-sectional, case-control, cohort, systematic reviews, meta-analyses, literature reviews, and randomized control trials. Articles written in other languages or ones that were published earlier than 2012 were not included.

Data Extraction

Independent reviews of the literature were done by two authors, who resolved any differences about inclusion through conversations and consensus. After reviewing the titles, the abstracts were examined to see whether the titles matched the criteria for inclusion. Abstracts that met the criteria for inclusion were read in full, and research that still met the criteria for inclusion underwent data extraction. Table 2 lists the accepted studies for this study and the quality assessment tools [1-3,11-13,16-22].

**Table 2:** Quality assessment of each type of study AMSTAR: Assessment of Multiple Systematic Reviews; AXIS: Appraisal Tool for Cross-Sectional Studies; NOS: Newcastle-Ottawa Scale; SANRA: Scale for the Assessment of Narrative Review Articles.

Quality assessment tool	Study type	Total score	Accepted score (>70%)	Accepted studies
AMSTAR [16]	Systematic review and meta-analysis	16	12	Stephan et al. [3]
AXIS [17]	Cross-sectional	20	14	Ghosh et al. [2], Crespi et al. [11], Fama et al. [12], Galandra et al. [13], Adhikari et al. [18]
NOS [19]	Cohort	8	6	Galandra et al. [1]
SANRA [20]	Literature review	12	9	Burnette et al. [21], Wilcox et al. [22]

Results

**Figure 1:**
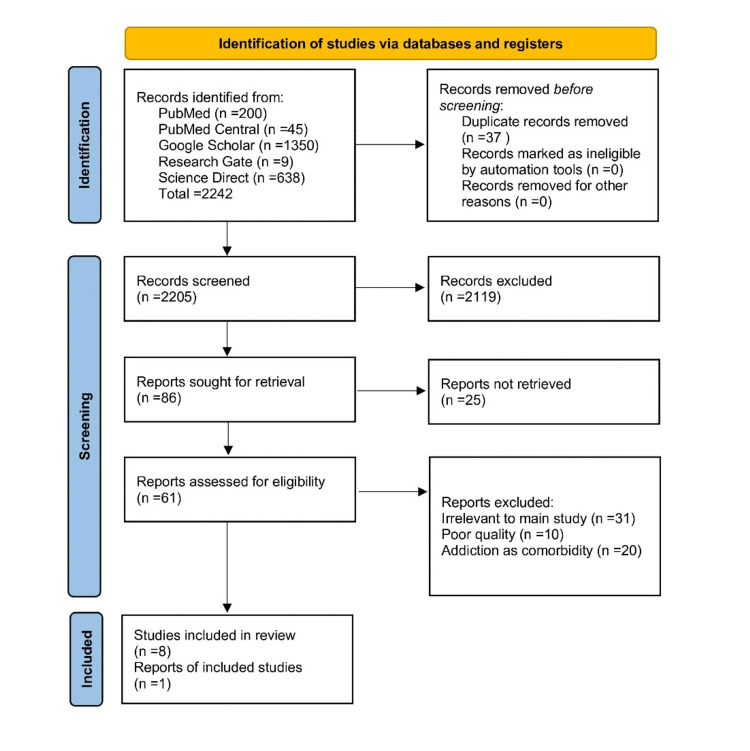
PRISMA flow diagram of literature search PRISMA: Preferred Reporting Items for Systematic Reviews and Meta-Analyses.

There were 2242 potentially related titles found in the database search. Using EndNote (Clarivate Analytics, Philadelphia, PA), 37 duplicates were identified and eliminated. When the titles and abstracts of these records were scrutinized using this review's PICO (Patient/Problem, Intervention, Comparison, and Outcome) elements and eligibility standards, only 86 papers remained. These articles were located, and each one underwent a quality evaluation. The PRISMA flow diagram and the search procedure utilized in this investigation are shown in Figure 1.

The systematic review included nine studies assessed as having high quality. Table 3 summarizes the studies selected for the systematic review.

**Table 3:** Summary of studies taken for systematic review AUD: alcohol use disorder; EF: executive functioning; GM: gray matter; ENB: Esame Neuropsicologico Breve; TMT-A: Trial Making Test A; TMT-B: Trial Making Test B; BART: Balloon Analogue Risk Task; rDLPFC: right dorsolateral prefrontal cortex.

Author	Study type	Year of study	Number of subjects	Conclusion
Galandra et al. [1]	Cohort	2019	41	Alcoholic patients' cognitive deficits are caused by functional mechanisms that go beyond the frontal lobes' specific susceptibility. Executive dysfunction in AUD patients appears to reflect altered connectivity in frontostriatal neural mechanisms that support cognitive control and top-down behavior modulation by mediating the transition between automatic and controlled processing.
Ghosh et al. [2]	Cross-sectional study	2018	100	Regular and ongoing alcohol use significantly impairs executive function. A regular neuropsychological evaluation is therefore crucial for the early identification and correction of underlying deficits, which completes the treatment of alcoholism.
Stephan et al. [3]	Meta-analysis	2017	77	By comparing the effect sizes between healthy comparison groups and detoxified subjects with AUD across the five composites of EF and three subcategories of impulsivity, the results of this meta-analysis suggest that planning, problem-solving, inhibition, and self-regulation—decisional and cognitive impulsivity more so than motor disinhibition—are severely impacted by alcohol abuse.
Crespi et al. [11]	Cross-sectional study	2020	40	The current findings thus pave the way for future basic and translational research, including identifying critical components of executive dysfunction in AUD to customize novel intervention strategies and integrating multi-modal imaging data to develop a comprehensive model of its anatomic-functional bases at various scales.
Fama et al. [12]	Cross-sectional Study	2019	131	These findings show that age and total alcohol consumption have different moderating effects on the cognitive and motor deficits associated with alcoholism.
Galandra et al. [13]	Cross-sectional study	2018	41	They observed a pattern of distributed GM density reduction in AUD patients who performed noticeably worse on the ENB global score and various sub-scores: immediate recall, interference memory-10, TMT-A and B, overlapping images, and clock drawing.
Adhikari et al. [18]	Cross-sectional study	2016	62	Long-term alcohol use is directly linked to cognitive function impairment, whereas higher education offers protective benefits, especially for executive functions.
Burnette et al. [21]	BART study	2020	32	Alcohol-dependent participants showed less rDLPFC activation modulation when faced with risk. Exploratory analyses found that during explosions in a cluster involving the insula, alcohol-dependent participants activated less than controls did.
Wilcox et al. [22]	Literature review	2014	This study mentions the study done by Li et al. (2009), Schmaal et al. (2013), where n = 16, Karch et al. (2008), and Scult et al. (2012)	Clinical results may be impacted by decreased cognitive control in AUD, particularly when there are abnormalities in inhibitory functioning.

Discussion

The concept of executive function needs to be deconstructed to comprehend the relative contribution of different cognitive executive processes to higher-order cognitive abilities like decision-making in AUD. Due to the complexity of these functions and the use of various tasks to evaluate various constellations of component processes, studies have found severe variability in executive function impairment. The majority of standard tasks used to evaluate executive functioning are multidimensional and involve several different processes that make up executive function [23].

Clinical Neuropsychological Tests of Executive Dysfunction

Day et al. (2015) provided detailed descriptions of the tasks [4]. Table 4 depicts the variety and quantity of tasks used to evaluate EF among drinkers following alcohol delivery.

**Table 4:** Tasks of executive functions Variety and quantity of tasks are used to evaluate executive functioning among drinkers following alcohol delivery [4]. WAIS-III: Wechsler Adult Intelligence Scale, Third Edition.

Task	Measures	Description
Wisconsin Card Sorting Test	Set shifting, rule acquisition	Examinees must acquire a flexible set of rules to classify the test stimuli based on examiner input appropriately.
Trail Making Test	Set shifting	Examinees must swiftly link a page's random assortment of numbers (Part A) or letters and numbers (Part B) in the correct sequence.
Mental arithmetic from WAIS-III	Working memory (verbal)	Examinees are given oral presentations of arithmetic word problems and are required to solve them without the aid of paper or a pencil.
Self-Ordered Pointing Task	Working memory (visuospatial), self-regulation	Examinees must point to variously arranged things without pointing twice to the same item.
Tower of London/Hanoi/tower test	Planning/inhibition	Move the discs or beads following a strict set of guidelines.
Iowa gambling task	Planning	Examinees must decide which deck gives the highest odds to maximize profits while dealing cards from different reward/penalty levels.
Go/No-Go	Response inhibition	A variety of tasks in which test subjects must respond to one stimulus while delaying their response to another stimulus.
Stop signal	Response inhibition	The aim variable is response time; test subjects must begin a motor sequence and terminate the activity at a signal.
Stroop task	Response inhibition; resistance to interference	The examinee is given a set of color names printed in inconsistent ink colors; they must disregard the words and pick out the ink colors as rapidly as possible.
Controlled Order Word Association Test (COWAT)	Mental flexibility; set maintenance	Examinees must quickly identify words that start with a target letter while avoiding proper nouns and suffix-related variations.

Other Tests

Emotion face expression (EFE): Given the significance of facial expressions in interpersonal and social interactions, several studies use modifications of EFE tasks to examine the accuracy of emotion processing. EFE exercises frequently demand participants to assess the kind and/or severity of the emotions depicted on facial stimuli [24].

Near transfer tests included the rotation span (RTS), the reading span (RDS), and the auditory consonant trigram (ACT). A letter is shown to participants in the RTS task either in the right direction or in a mirror-reversed way. After the letter has been rotated to one of eight different angles, participants are asked to choose whether the letter is facing the proper or mirror-reversed direction when turned upright. Participants see a short or long arrow pointing in one of the eight directions after making their decision. The participants are then instructed to click on the arrows that were displayed in the proper sequence after a series of judgments and arrows. After every set of trials, participants are asked to recollect the consonant strings, much like in the other near-transfer tasks. Each task (RTS, RDS, and ACT) requires the subject to remember information while executing secondary distracting tasks, which taps the attentional-control mechanisms of the central executive of white matter (WM) capability [25].

The running letter span (RLS), running spatial span (RSS), and keep track (KT) activities are used to evaluate the minor transfer effects of training on working memory capacity. A series of letters are presented one after another in the RLS challenge (two letters per second). Participants must memorize a set size (the number of letters they must remember) prior to the presentation and recollect the most recent set size (n) in the proper serial position. For instance, participants might get seven letters after being instructed to recall the prior five delivered letters. Set sizes ranged from three to nine, with each trial comprising two trials [25].

Specific Brain Regions Most Affected by Alcohol Neurotoxic Effects

Conceptualization, programming, and mental flexibility, which were more adversely affected by chronic alcohol consumption, have been connected to the operation of the medial, dorsolateral, and posterior regions of the prefrontal cortex [18]. A PET study found GM shrinkage in the frontostriatal, frontocerebellar, and frontolimbic circuit nodes, including the frontal and cerebellar cortices, cingulate gyrus, thalamus, and hippocampus [7]. In vivo MRI and postmortem neuropathological studies of uncomplicated alcoholics showed that the frontal lobes display the most apparent cortical abnormalities, with concurrent corpus callosum thinning and concurrent compromise of the pontocerebellar and cerebello-thalamo-cortical systems [15].

A study done by Fama et al. on alcohol dependents and controls showed that precentral frontal and hippocampal sizes were smaller in AUD than in controls, and they had worse scores on all memory tests. The orbital, superior, and inferior frontal volumes, and lifetime alcohol consumption, were independent predictors of episodic memory in AUD. A two-fold dissociation was used to establish selectivity; it showed that inferior frontal volumes predicted visual but not verbal memory, whereas orbital frontal volumes predicted both. Furthermore, orbital frontal volumes predicted verbal memory in AUD + drug addiction history, whereas superior frontal volumes predicted verbal memory in AUD alone [12,25]. VBM studies have in fact shown atrophic patterns in AUDs in the lateral prefrontal cortex, anterior and posterior cingulate cortex, insular-opercular cortex, thalamus, hippocampus, and striatum [12,13]. These data support the idea that the executive and salience networks, which serve as key nodes in the cortico-striatal-thalamic circuit and mediate the transition between relaxed and controlled cognition and behavior, are involved in the cognitive changes seen in alcohol addiction [13].

The mesocorticolimbic pathways: Subcortical (the ventral striatum, thalamus, hippocampus, and amygdala) and cortical (the ventromedial and posterior dorsomedial frontal cortex) components showed a broad pattern of GM loss in patients. An impairment of this route has been proposed to underlie the development and maintenance of addiction, via negative reinforcement, based on fMRI evidence for its role in adaptive behavioral learning. The primary nodes of the so-called "salience network," the dorsal anterior cingulate cortex (dACC) and insular cortex, also exhibited GM atrophy [18]. The idea that the cognitive and behavioral changes seen in AUDs may represent functional imbalances within a cortico-striato-thalamo-cortical regulatory loop supporting executive control and self-regulation accords with this evidence of extensive brain damage [13].

A study done by Crespi et al. used whole-brain voxel-wise analyses of diffuse tensor imaging measurements to assess WM microstructural deterioration and its relationship with fundamental executive dysfunction in AUD patients compared with healthy controls. It found a consistent pattern of WM microstructural deterioration in all the investigated diffusivity indices [11]. It was possible to link the impairment, which mainly affects psychomotor speed, working memory, and attention, to various aspects of microstructural modification in different bundles, thanks to a comprehensive characterization of WM damage in AUD patients. The extent of microstructural deterioration at the global (whole-brain) and local (corpus callosum) scales, which may be regarded as a general marker of illness history, was correlated with the length of alcohol misuse [11].

Studies using neuroimaging techniques to uncover the brain correlates of EF deficiencies may be better able to detect minute changes in EF than studies using solely traditional EF tests. Therefore, neuroimaging research may help us better understand how subtle EF deficits and alcohol use behaviors are related. It may also help us spot people who are at risk of developing AUDs [9]. The frontostriatal, frontolimbic, and frontocerebellar neural systems, which extend to subcortical and cerebellar structures and are connected, have recently been identified as three independent yet highly interconnected frontally based neural systems affected in chronic alcoholism. These neural systems are linked to alcoholism-related cognitive and motor deficits [12].

Neurophysiological Correlates of Alcohol Use Disorder

An increasing body of research indicates that people with AUD who have just quit drinking have poor emotional facial expression (EFE) processing. Anger processing was more severely impacted than other emotions, and it was linked to interpersonal issues such as being too reliant, passive, and accommodating. The lack of sufficient data in this area highlights the necessity for in-depth research on emotion processing and its practical applications [26]. These cognitive deficits not only affect daily management but also significantly affect management effectiveness. Therefore, it seems crucial to recognize cognitive impairments brought on by alcohol so that alcohol treatment can be modified to meet their requirements [18]. In addition to the effects mentioned above, excessive alcohol use can also lead to several other issues, such as the use of other drugs or alcohol, the loss of a job, domestic or interpersonal violence, cancer, cirrhosis, and mental health issues [6]. There have been several studies on the subject, with neurobiological studies taking center stage in recent years [2]. Teenagers who abuse alcohol are more prone to develop internet addiction disorder and exhibit specific psychological traits, such as high behavioral activation, low self-esteem, low family function, and life satisfaction [14].

Understanding individual variability in EF is essential for an understanding of the causes, effects, and treatment of AUDs [9]. For instance, difficulties with set-shifting or knowledge updating may make it more difficult for someone to employ various coping strategies when faced with alcohol-related stimuli. A person's potential to resist the urge to visit a bar, hang out with people who drink, or consume alcohol may also be impacted by difficulties with response inhibition. The wide range of EF processes that have been studied about alcohol use provides evidence of this complexity. As mentioned above, while some studies use scores on a single task that only evaluates one process, others use scores on a composite score that is built from numerous tasks. Despite the differences across these studies, alcohol appears to have acute effects on various EF functions, such as updating, set-shifting, and response inhibition. However, some EF functions (such as verbal and auditory working memory) are influenced by alcohol more consistently than others (visuospatial working memory is not as reliably affected) [9].

**Figure 2:**
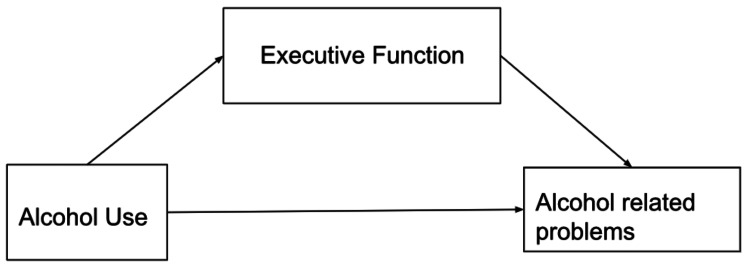
The mediation role of executive function on the connection between alcohol consumption and alcohol-related disorders Image modified from the article by Powell et al. (2021) [28].

Large-scale cognitive impairments are linked to alcohol dependence, especially for executive functions. These deficits are still present even after decades of abstinence, significantly affecting patients' day-to-day functioning and risk of relapse [27]. The nature of EF deficits in risky drinking and the mediating impact of EF and drinking on real-world functioning suggest that risky drinkers may be more vulnerable [28]. The relationship between alcohol use and alcohol-related problems was partially mediated by the effect of alcohol use on subjective EF, showing the importance of understanding and addressing poorer EF in hazardous drinkers. Hazardous drinkers reported significantly lower subjective EF. Additional methods, such as the recovery of function, should be used in future studies to assess EF in hazardous drinking. These methods should also be used to determine which alcohol-related issues EF mediates and to consider possible interventions [28]. Figure 2 depicts the mediation role of executive function on the connection between alcohol consumption and alcohol-related disorders [28].

Chronic alcoholism impairs neuropsychological functions, which may hinder clinical treatment effectiveness (e.g., cognitive behavioral therapy, which requires learning new complex knowledge) and potentially obstruct efforts to enable other higher-order abilities like successful interpersonal and social interactions, non-risky decision-making, and awareness of cognitive and behavioral dysfunctions (i.e., accurate metacognition) [25]. Drug acquisition, escalation/dysregulation, abstinence, and relapse are all stages of the addiction process that significantly rely on the decisional and cognitive components of impulsivity [25]. Le Berre et al. (2017) showed the correlation between chronic drinking and various factors interconnected with the inability to abstain from alcohol [23].

A review done by Zilverstand et al. (2018) showed persistent associations between alcohol and other drug use and activation levels of the reward and executive networks, with reward network activation being positively correlated with alcohol (and any other drug of abuse investigated) and recruitment of the executive network being negatively correlated, which were linked to increased addiction severity and inability to remain abstinent [24].

Long-term alcohol use significantly impairs conceptualizing, programming, inhibitory control, and general executive function. Poor executive functioning was correlated with more extended periods of alcohol use and dependency [18]. Of individuals with alcoholism, 33.9% had poor performance in several executive function areas. The majority of the research participants had memory, logical reasoning, problem-solving, and cognitive flexibility impairments, making concept identification a challenging task [18]. Alcohol-related executive deficits go beyond the inhibition impairment that is typically mentioned. Each EF subcomponent is affected by this impairment, as alcohol-dependent individuals displayed more severe deficits in updating and shifting skills. This initial discovery of a complex EF deficit highlights the necessity of an individual EF assessment and rehabilitation procedure during and/or after the detoxification procedure [27]. The most damaged capabilities among alcohol addicts were visual retention and quick recall, according to recent research [18]. Working memory impairment in both verbal and nonverbal domains was correlated with lifetime alcohol use, suggesting a dosage impact of alcohol addiction. As opposed to verbal memories, visual memories are also affected by alcohol consumption over more extended periods [18].

Alcohol-Related Dementia

Additionally, it is clear that some alcoholics exhibit clinically significant neurocognitive impairment but do not fully fit the bill for Wernicke-Korsakoff syndrome (WKS) or other distinct alcohol-related neurological disorders (like Marchiafava-Bignami disorder). Alcohol-related dementia (ARD) has been suggested as a diagnosis for those who are more impaired within this group, although there has been some debate over this. Although ARD is described as having a more gradual global decline in cognition than WKS, the choice of name has drawn criticism because, unlike most dementias, the disorder does not worsen with abstinence [29].

In place of other diagnostic nomenclature, the term "alcohol-related brain damage" (ARBD) has recently been adopted to describe a spectrum of neurocognitive impairment that includes both WKS and ARD. By acknowledging the varied effects of chronic alcoholism and its contributing factors, this emerging conceptualization may offer a more practical nosological approach (e.g., nutritional deficiencies, hepatic dysfunction, cerebrovascular disorders, and head injury) [29]. There is disagreement regarding the etiopathogenesis of the disorder as well. One school of thought links ARD to the direct neurotoxic effects of alcohol, such as neuroinflammation and glutamate excitotoxicity that occur during withdrawal, while another contends that ARD is merely a variant of WKS brought on by thiamine depletion [29].

Limitations

Our research has some limitations. We only selected articles in the English language and published articles; several research studies we chose had tiny sample sizes and could not accurately represent the population as a whole. We only included research published after 2012; however, some earlier studies may contain crucial findings. The results of the studies using MRI scans would have contributed to a better knowledge of the damaged areas, the volume of the brain, and connections. This study will aid future researchers by providing clarification and opening up new avenues for investigating the elements of executive dysfunction in AUD.

## Conclusions

Alcohol-dependent individuals have serious memory and executive function problems in several areas, and decisional and cognitive impulsivity, problem-solving, inhibition, and self-regulation are affected. Long-term alcohol use is closely linked to cognitive function degradation, but greater education offers protective benefits, especially for executive skills. Therefore, regular cognitive testing as part of an alcohol treatment program may help identify and monitor the development of these changes as well as for cognitive rehabilitation and psychosocial reintegration of alcohol dependents. Improved treatment results continue to be a major public health priority because of the enormous amount of suffering and expense associated with alcohol addiction problems. A promising method that could promote treatment compliance and lessen problematic alcohol consumption is cognitive rehabilitation, particularly in the crucial and complex domain of executive functioning. The conclusions discussed above need to be supported by more studies, and neuropsychology research for cognitive deficiencies has to be prioritized.
